# Supplementary material for: Inflammatory factors and risk of lung adenocarcinoma: a Mendelian randomization study mediated by blood metabolites
Source: Front Endocrinol (Lausanne). 2024 Aug 27;15:1446863. doi: 10.3389/fendo.2024.1446863 (PMC11384989; doi:10.3389/fendo.2024.1446863)
Supplement: Supplementary file 1 [file DataSheet1.zip › supplementary files/S2/GCST90200413/sensitivity-analysis.pdf]

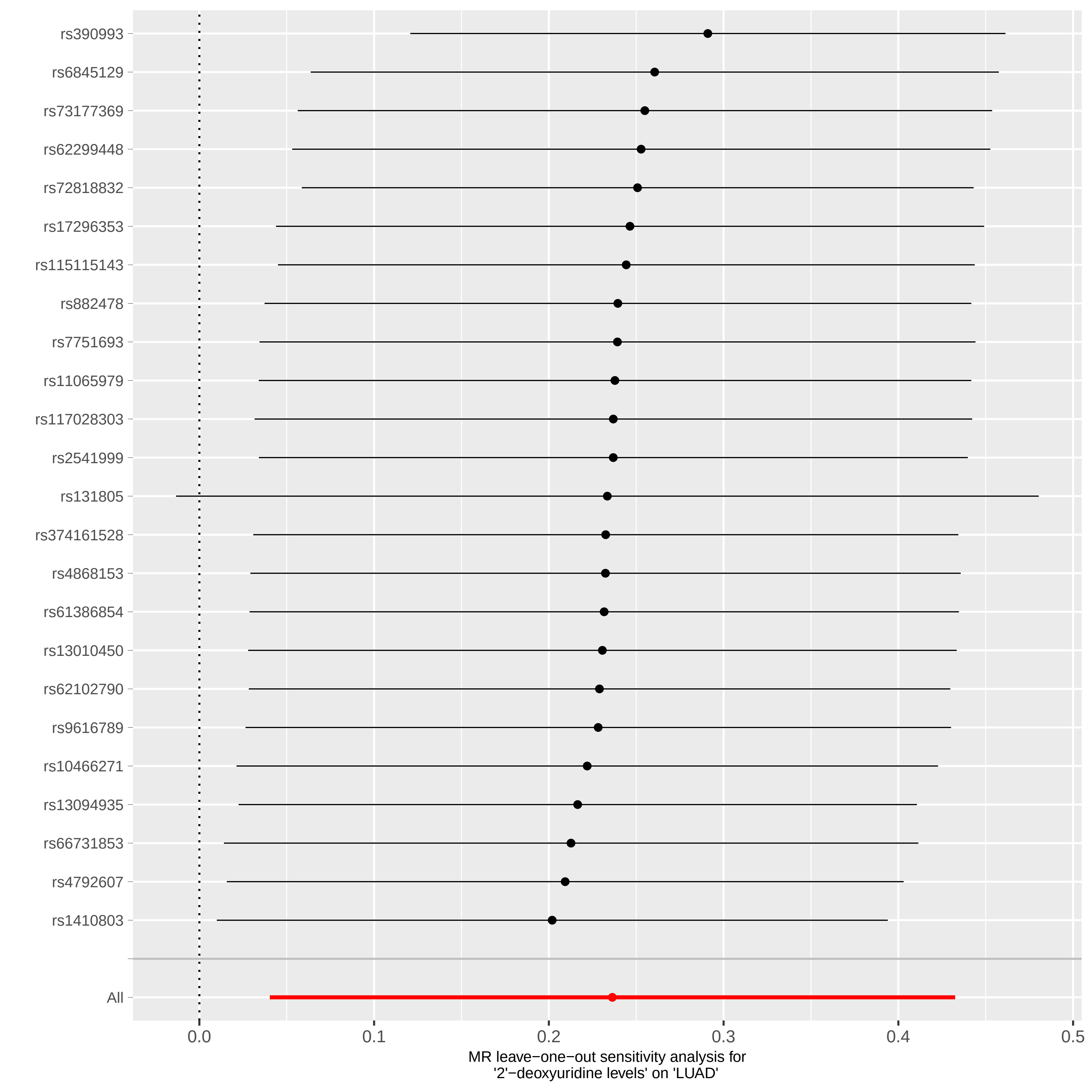

rs390993
rs6845129
rs73177369
rs62299448
rs72818832
rs17296353
rs115115143
rs882478
rs7751693
rs11065979
rs117028303
rs2541999
rs131805
rs374161528
rs4868153
rs61386854
rs13010450
rs62102790
rs9616789
rs10466271
rs13094935
rs66731853
rs4792607
rs1410803
All
0.0
0.1
0.2
0.3
0.4
0.5
MR leave−one−out sensitivity analysis for
'2'−deoxyuridine levels' on 'LUAD'
